# Photocurable Nanocellulose-Based Hydrogel for Real-Time Electrochemical Sweat Monitoring in Smart Textiles

**DOI:** 10.1021/acs.langmuir.5c04977

**Published:** 2025-12-15

**Authors:** Kai-Wen Chuang, Po-Kuan Li, Yun-An Huang, Ying-Chih Liao

**Affiliations:** Department of Chemical Engineering, 33561 National Taiwan University, Taipei 10617, Taiwan

## Abstract

Wearable sweat sensors demand hydrogels that wet textiles rapidly, cure on demand, and retain mechanical integrity during use. In this work, a UV-curable poly­(acrylic acid)/cellulose nanocrystal (PAA/CNC) hydrogel was developed to improve the performance and reliability of flexible electronic sensing systems. The formulation gels within about 2 to 4 s across the series and forms a uniform network suitable for scalable coating and patterning. CNCs simultaneously enhance hydrophilicity and mechanical robustness: the static contact angle decreases from 37.1° at 0 wt % CNC to 9.9° at 10 wt % CNC, while the equilibrium swelling ratio peaks at 657% for 8 wt % CNC and remains high at 650% for 10 wt % CNC, balancing hydrations with dimensional stability. Bulk properties improve with CNC loading, with maximum tensile strength increasing from 133 to 180.7 kPa and Young’s modulus from 0.73 to 1.25 MPa, followed by a modest plateau beyond about 5 to 8 wt % that is consistent with emerging filler–filler interactions. FTIR indicates esterification between PAA and CNC, and SEM reveals a continuous porous morphology favorable for ion transport. Overall, the 10 wt % CNC formulation maximizes stiffness and strength while maintaining good hydrophilicity and rapid photocuring behavior. These attributes position photocured PAA/CNC hydrogels as promising matrices for textile-integrated, impedance-based sweat sensing.

## Graphical abstract

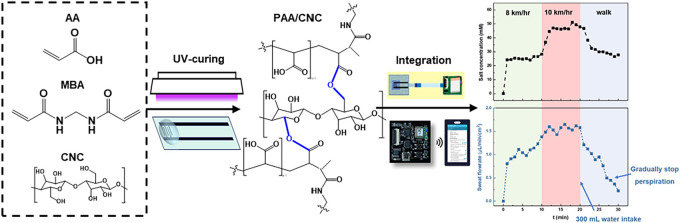

## 1 Introduction

In the era of rapid technological advancement, the Internet of Things (IoT) has catalyzed the development of intelligent wearable devices, particularly in the field of health monitoring.^1,2^ Among these, smart textiles that integrate electronic functionality into fabrics have shown immense potential by enabling sensing, signal transmission, and light emission within everyday clothing.^1,3^ Wearable biosensors,^4^ in particular, have attracted considerable interest for their ability to provide real-time physiological data through noninvasive means such as sweat analysis.^5^ Yet, realizing fully integrated sweat-sensing textiles^6,7^ remains challenging because the textile environment imposes cyclic bending, shear, and laundering that degrade sensor layers and interconnects, and because the skin–sweat–fabric interface is mechanically and chemically dynamic. These factors make long-term stability, washability, and comfort decisive hurdles for adoption, not merely performance, after initial fabrication.

At the molecular scale, the chemistry of poly­(acrylic acid) (PAA) presents inherent instability. Its carboxyl groups ionize differently across the physiological sweat pH range (4.5–7.0), altering polymer chain repulsion, swelling ratio, and conductivity. This pH sensitivity causes the hydrogel’s electrochemical response to drift over time and complicates calibration.^4,8^ Moreover, heterogeneity in ionization across the polymer matrix creates spatially nonuniform charge distributions, which propagate into local conductivity gradients. Such instabilities undermine the reproducibility that is essential for quantitative biosensing.

At the material scale, swelling remains a fundamental limitation. Water uptake drastically softens PAA-based hydrogels, reducing modulus and weakening shear strength at the film–fiber boundary.^9^ This creates a fragile balance between cohesion within the hydrogel and adhesion to textile fibers: when adhesion is weaker, peeling at the interface dominates; when cohesion is weaker, the bulk hydrogel fractures.^10^ Attempts to reinforce one failure mode often exacerbate the other, reflecting the intrinsic trade-off between cohesion and adhesion in soft hydrogel–textile hybrids. Compounding this, wetting behavior on woven or knitted fabrics is highly inconsistent: heterogeneous pore structures and surface roughness generate contact-angle hysteresis, resulting in irregular electrolyte penetration and incomplete electrode coverage. These variations translate into unstable ion transport pathways and erratic electrochemical impedance signals.

Complicating matters further are the processing limitations associated with conventional thermal curing.^11^ Because swelling and adhesion already strain material performance, the introduction of heat-driven polymerization amplifies heterogeneity. Elevated curing temperatures and extended reaction times accelerate solvent evaporation, producing gradients in cross-link density and leading to brittle or uneven films. These effects undermine both the ionic conductivity and the mechanical resilience. In addition, thermal curing is poorly suited to temperature-sensitive substrates, such as textiles and elastomers, where shrinkage, warping, or delamination can occur during processing. The reliance on batchwise curing also restricts scalability, making it difficult to adapt hydrogel fabrication to high-throughput methods such as roll-to-roll processing and digital patterning.

At the textile and device scales, these microscopic and processing weaknesses accumulate into macroscopic failure. Conventional integration strategies^12^—including conductive fiber weaving, screen printing, and vapor deposition—struggle to simultaneously achieve durability and scalable throughput.^13^ Printed metallic inks, for instance, demand multiple deposition passes to achieve acceptable conductivity but remain intrinsically brittle, showing sharp resistance increases under bending, creasing, and laundering. Hydrogels adhered to textiles are similarly vulnerable: cyclic deformation^14^ accelerates delamination, while repeated laundering promotes swelling-induced drift, mechanical fatigue, and signal loss. Furthermore, even when electrodes remain functional, electrochemical sweat sensors are prone to baseline drift and require periodic recalibration due to surface fouling and gradual activity loss. Advanced device platforms, such as microfluidic epidermal patches,^5^ and screen-printed textiles,^15^ expand functional possibilities but introduce their own drawbacks, including rigid microchannel architectures, unstable performance after repeated washing, and wearer discomfort arising from multilayer encapsulation or brittle substrates.

In this study, a photocurable PAA/CNC hydrogel film^16,17^ is developed that directly addresses these challenges. Photoinitiated curing enables ultrafast gelation within seconds, preserving water content and ensuring uniform film formation, while CNCs provide nanoscale reinforcement via hydrogen bonding^18^ and chain entanglement.^15^ Darocur 1173 was selected for its high acrylate conversion efficiency,^19^ biodegradability,^20^ and rapid cross-linking at low loading, outperforming alternatives such as TPO. Cooperation of CNC further yields a mechanically resilient, highly wettable, and strongly adherent coating that couples seamlessly with textile substrates. Integrated with printed interdigitated electrodes^21^ and a lightweight system-on-chip for impedance-based sensing,^22^ the platform captures sweat volume and ion concentration in real time while maintaining structural integrity under repeated deformation and immersion (Figure 1). By combining scalable photochemistry with nanoscale reinforcement, the approach bridges the gap between sensitivity and durability in textile biosensing—offering not just a functional coating but a generalizable strategy for intelligent and comfortable fabrics capable of long-term physiological monitoring.

**Figure 1:**
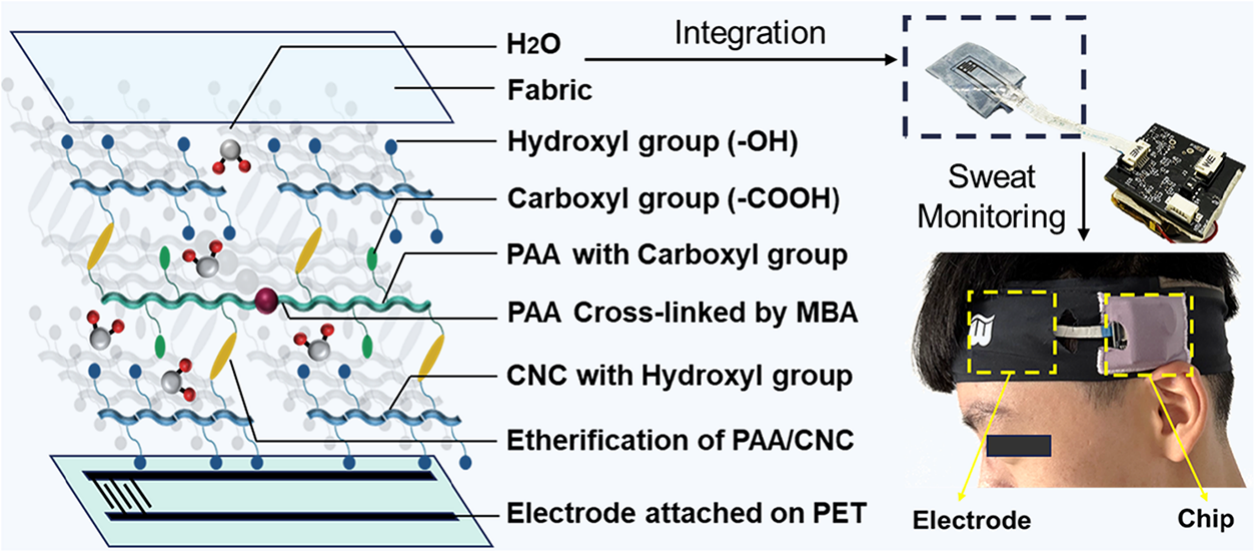
Schematic illustration of the UV-cured PAA/CNC hydrogel network and its integration with a wearable sweat-sensing system for real-time flow rate and ionic concentration monitoring.

## 2 Experimental Section

### 2.1 Material

Acrylic acid (AA, 98%) was purchased from Thermo Scientific. High-purity deionized (DI) water was sourced from Merck Millipore with a resistance greater than 18.2 MΩ/cm. Polyethylene terephthalate (PET) sheets were supplied by Cymmetrik Enterprise. Cellulose nanocrystals (CNCs) were sourced from CelluForce. *N*,*N*′-Methylenebis­(acrylamide) (MBA, ≥99.5%) was supplied by Sigma-Aldrich. The photoinitiator 2-hydroxy-2-methylpropiophenone (Darocur1173) was obtained from Emperor Chemical. Sodium chloride (NaCl, 99.5%) was provided by ACROS Organics. Double-sided heat-fusible adhesive film was purchased from Dahe Industry Technology.

### 2.2 Hydrogel Preparation—Formation of PAA/x-CNC

The formation of PAA/x-CNC hydrogels began with the separate preparation of PAA and CNC solutions. First, 2.5 g of acrylic acid (AA) and 0.025 g of the photoinitiator Darocur 1173 were mixed using a planetary centrifugal mixer (ARE-310, THINKY) at 2000 rpm for 3 min to ensure complete dissolution of the initiator. In parallel, the desired number of cellulose nanocrystals (CNCs) was combined with 0.05 g of *N*,*N*-methylenebis­(acrylamide) (MBA) and 7.3 g of deionized water, followed by homogenization under the same mixing conditions (2000 rpm for 4 min) to achieve uniform CNC dispersion. The prepared CNC/MBA solution was then mixed with the AA/initiator solution, and the resulting precursor was further homogenized and subsequently defoamed at 2200 rpm for 1 min to remove entrapped air bubbles. These procedures produced five distinct photocurable formulations: PAA/10CNC, PAA/8CNC, PAA/5CNC, PAA/2CNC, and PAA/0CNC, corresponding to CNC concentrations in descending order.

For film fabrication, the prepolymer mixture was deposited directly onto the interdigitated electrodes (IDEs) to form a uniform coating. Prior to deposition, the electrode surface was sequentially cleaned with deionized water, ethanol, and acetone, then treated with an atmospheric plasma system for 5 min to enhance wettability and adhesion. The hydrogel precursor was pipetted onto the IDE surface and evenly spread via spin coating (SP-M1-S, Collect) at 500 rpm for 10 s, followed by 1000 rpm for 30 s. Finally, the coated films were exposed to 345 nm UV light in a UV curing box (TOPBI 2020-2) for 1 min to initiate photopolymerization and complete network cross-linking, yielding the final PAA/CNC hydrogel films.

### 2.3 Characterizations

Curing kinetics were monitored using a TA Discovery HR-2 rotational rheometer by simultaneously recording the storage modulus (G′), loss modulus (G″), and viscosity evolution of the photocurable precursor under UV exposure. The gelation time was determined from the modulus crossover point (G′ = G″) and corroborated by the sharp rise in viscosity, both of which indicate the transition from a liquid-like state to a cross-linked hydrogel network during UV-induced polymerization and curing. Rheological measurements were conducted using a 20 mm parallel-plate geometry with a 1.0 mm gap at 25 °C under constant strain (1%) and angular frequency (1 rad s^–1^) to ensure consistent curing conditions. Shear stress (SS) and elongation at break (EAB) for all samples are measured using a universal testing machine JSV-H1000 (Japan Instrument System Co., Ltd., Japan) using an initial grip separation of 30 mm and a crosshead speed of 5 mm/min, in accordance with ISO 527-2. The functional groups of hydrogels before and after cross-linking were analyzed using Fourier transform infrared spectroscopy (FTIR, model Spectrum 100, PerkinElmer). The surface morphology of PAA/CNC hydrogels was examined by scanning electron microscopy (SEM, model S-4801, Hitachi). Prior to imaging, the hydrogels were immersed in water for 48 h to achieve full swelling and then freeze-dried using liquid nitrogen to preserve their porous morphology. The surface hydrophilicity of the PAA/CNC hydrogels was characterized by contact angle (CA) measurements using an in-house goniometer system with an Olympus camera (model Stylu). The swelling ratio of the hydrogels was determined by drying the samples at 70 °C to a constant dry weight (*W*_d_), followed by immersion in deionized water until equilibrium. The swollen weight (*W*_s_) was recorded, and the swelling ratio was calculated using the following equation:

1 $$swelling\;ratio=\frac{W_{s}-W_{d}}{W_{d}}\times 100\%$$

## 3 Results and Discussion

### 3.1 Photocured Hydrogel Network

The photocurable precursor inks can efficiently produce a hydrophilic hydrogel with uniformly incorporated CNC reinforcement after photocuring. In Scheme 1, the precursor mixture of AA, MBA, and CNC rapidly undergoes simultaneous free-radical photopolymerization and esterification under UV irradiation.^23^ The photoinitiator (Darocur 1173) decomposes to generate radicals that initiate AA chain growth to form PAA, while MBA covalently cross-links adjacent PAA chains via its terminal vinyl groups, producing a stable three-dimensional hydrogel under ambient conditions. During the same period, the carboxylic acid groups (−COOH) of PAA interact with the hydroxyl groups (−OH) on CNC surfaces through esterification, forming covalent PAA–CNC linkages that enhance network integrity. The two reactions proceed concurrently but at different rates. The photopolymerization dominates the initial second-scale curing, while esterification continues more slowly, reinforcing interfacial bonding during and after curing. This photopolymerization process coupled with covalent ester formation combines the water affinity of PAA with the mechanical reinforcement of CNC, achieving rapid and uniform network formation without the application of elevated curing temperatures.

**Scheme 1:**
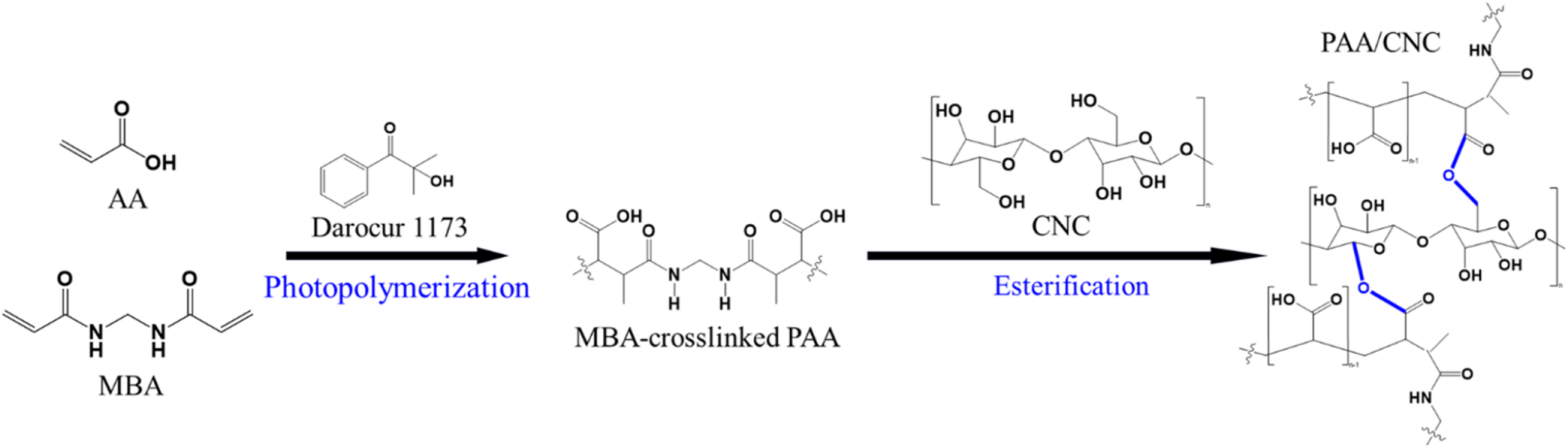
Mechanism of Simultaneous Photopolymerization and Esterification during the UV-Induced Cross-Linking Process

In Figure 2a, the weak absorptions between 815 and 950 cm^–1^, attributed to the vinyl (C=C) groups of acrylic acid (AA), appear in both PAA and MBA-cross-linked PAA before curing. Their disappearance after UV exposure indicates that the photocuring process effectively consumed the double bonds, even when the multifunctional cross-linker MBA was introduced. The band observed between 1690 and 1760 cm^–1^ corresponds to the C=O stretching of carboxylic groups, confirming their presence prior to curing. Although PAA itself shows an O–H stretching band, the incorporation of CNC increases the intensity and breadth of the signal in the 2500–3500 cm^–1^ region, suggesting enhanced hydrophilicity due to the hydroxyl groups of CNC.

**Figure 2:**
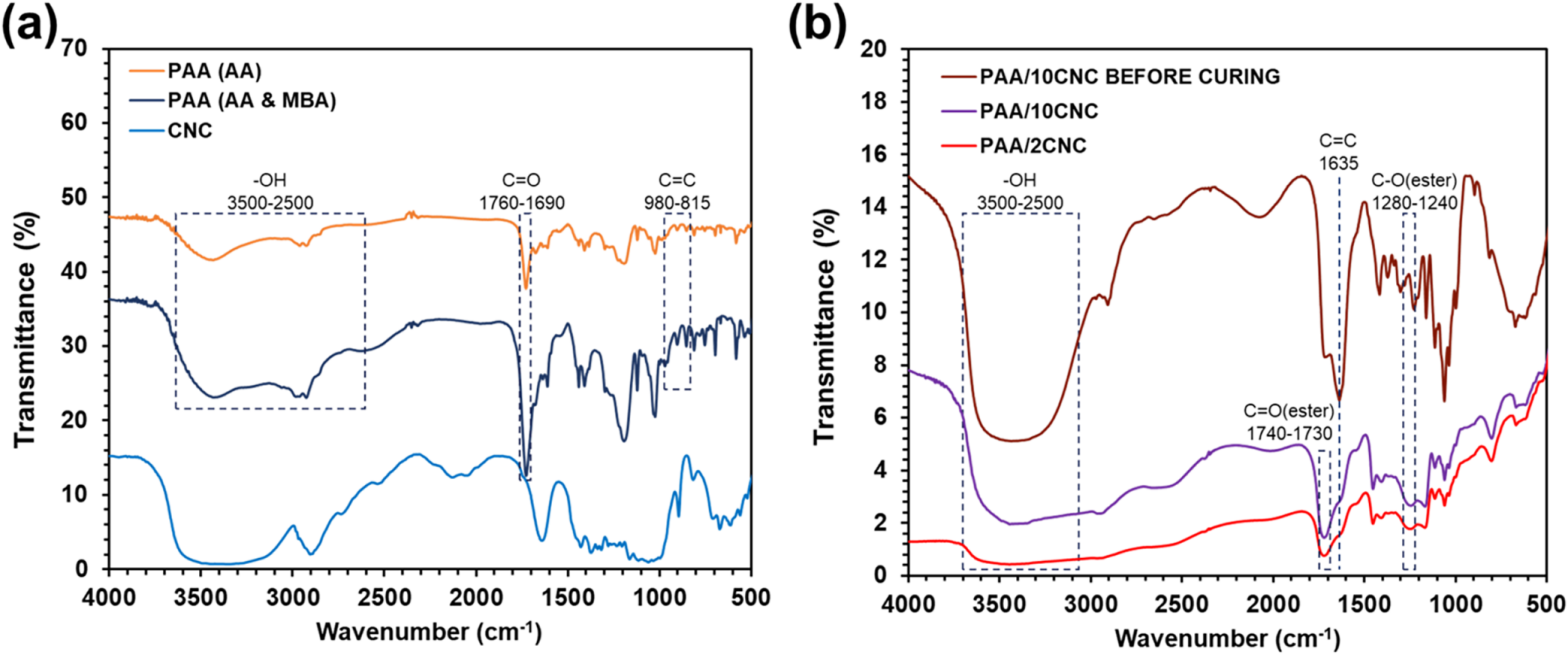
FTIR spectroscopy. (a) FTIR spectra of AA-based PAA, MBA-cross-linked PAA, and CNC. (b) FTIR spectra of PAA/CNC hydrogels before and after photocuring.

As shown in Figure 2b, the spectral changes further confirm the occurrence of both the photocuring and esterification processes. The distinct disappearance of the absorption band at 1635 cm^–1^ indicates that the C=C bonds of the vinyl groups were consumed during the photocuring reaction. In addition, the appearance of a new band at 1240–1280 cm^–1^, corresponding to the C–O stretching vibration of ester bonds, and the emergence of a C=O stretching band at 1730–1740 cm^–1^, which is absent in pure CNC, verify the formation of ester linkages through Fischer esterification between the carboxyl groups of PAA and the hydroxyl groups of CNC. Because part of the hydroxyl groups in CNC is consumed during this reaction, maintaining an appropriate CNC content is essential to balance hydrophilicity and cross-linking. The broad O–H stretching band near 3500 cm^–1^ remains evident in both uncured PAA/CNC and cured PAA/10CNC samples, whereas the PAA/2CNC sample exhibits a weaker and smoother O–H signal, reflecting a higher degree of esterification but still retaining a moderate level of surface hydrophilicity.

### 3.2 Rheological and Mechanical Properties of Hydrogel

As shown in Figure 3a, the PAA/CNC formulations undergo ultrafast gelation upon UV initiation, with the transition to a solid-like hydrogel occurring within approximately 5 s. When CNCs are incorporated, the gelation point shifts slightly, showing a modest increase in curing time compared with the CNC-free system (Figure S2). This delay is not indicative of poor reactivity but rather reflects the role of CNCs in altering the microenvironment of the network. The high surface area of CNCs can interact with polymer chains and partially restrict the mobility of reactive groups, slowing down chain motion during curing. At the same time, these interactions promote additional physical junctions that enhance the final cross-link density once the system is fully cured. Thus, the presence of CNCs balances an initial kinetic hindrance with a structural reinforcement effect, resulting in a network that is both robust and resilient. Such rapid yet reinforced curing ensures consistent mechanical integrity during scalable fabrication processes, while minimizing issues such as water loss or film heterogeneity, which are often problematic in thermally cured or slowly reacting systems. These properties make the formulation highly compatible with textile-based sensor integration and other scalable manufacturing routes.

**Figure 3:**
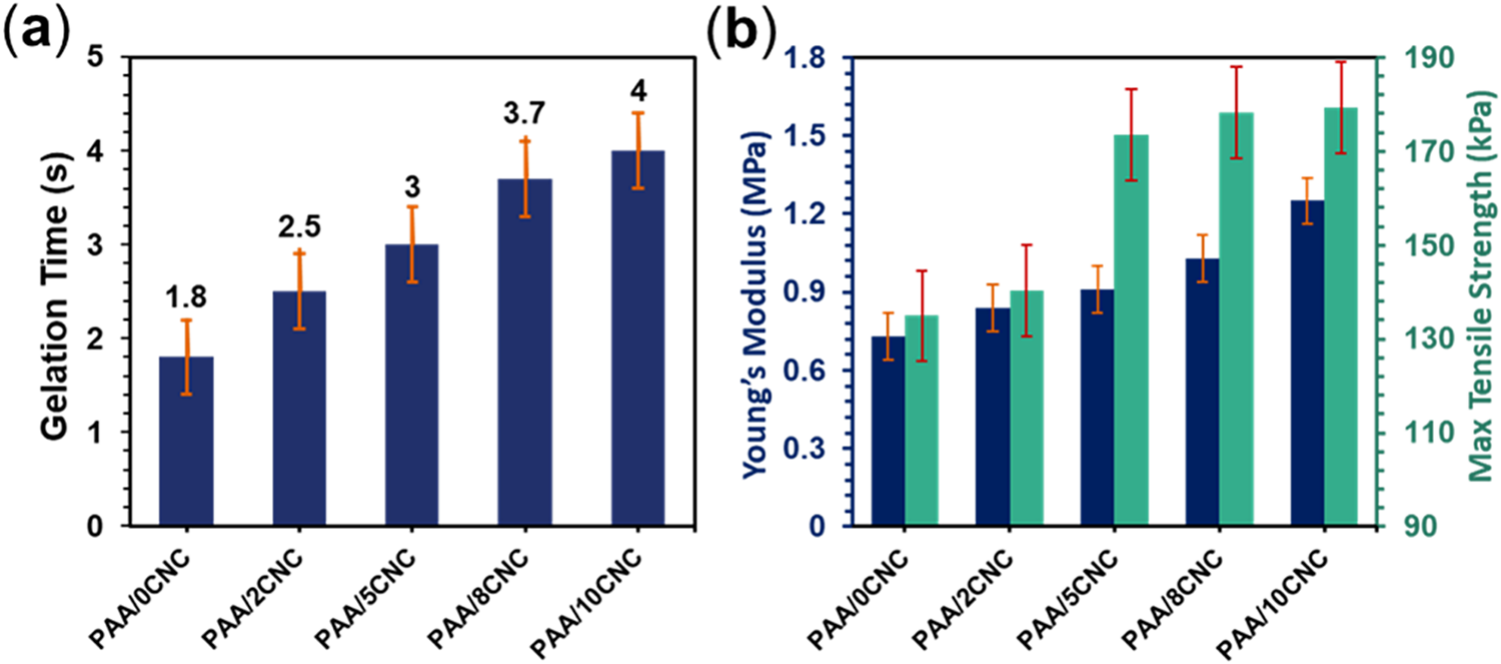
Rheological and mechanical properties of PAA/CNC hydrogels. (a) Gelation time at varying CNC contents. (b) Young’s modulus and maximum shear stress–strain showing improved strength with CNC incorporation.

As CNC loading increases, the hydrogel stiffens and strengthens in lockstep, reflecting more effective load transfer through the network (Figure 3b). Young’s modulus climbs from 0.73 MPa at 0 wt % CNC to 0.84, 0.91, and 1.03 MPa at 1, 3, and 5 wt %, reaching 1.25 MPa at 10 wt %, which is an overall gain of roughly 71% relative to the baseline. Maximum tensile strength follows the same monotonic trajectory, attaining its highest value at 10 wt % CNC. Notably, the slope of improvement tapers beyond 5 wt %, a hallmark of diminishing returns as filler–filler contacts and nascent aggregation begin to compete with polymer–filler interfacial bonding. In practical terms, these data show that modest CNC additions (3–5 wt %) already deliver substantial mechanical benefits with good efficiency, while higher loadings push absolute stiffness and strength to their maxima but with less incremental payoff. The trend is entirely consistent with a nanofiller-reinforced network in which CNCs act as rigid, hydrogen-bonding domains that constrain chain mobility and distribute stress until interparticle interactions saturate the available interfacial area.

### 3.3 Surface Wetting and Water Uptake Capacity

SEM analyses (Figure 4a) further validate the enhanced hydrophilicity imparted by CNC incorporation and ultrafast photocuring. The PAA/CNC network exhibits a sponge-like morphology with uniformly distributed pores, indicative of efficient radical propagation and homogeneous cross-linking. These interconnected pores serve as water reservoirs, promoting hydration and ionic transport. By contrast, CNC-free PAA (PAA/0CNC) presents a compact, densely packed morphology with a limited free volume for water infiltration. As the CNC content increases, the pores become more visible and slightly larger, suggesting that CNC not only enhances hydrophilicity but also facilitates the formation of interconnected microvoids that favor water diffusion through the porous network. This is mainly because the abundant hydroxyl groups on CNC form strong hydrogen bonds with the polymer chains, locally disturbing the curing uniformity and inducing microphase separation, which in turn promotes the generation and expansion of pores within the matrix. This morphological trend also correlates well with the swelling behavior (Figure 4d), confirming that CNC improves both surface wettability and bulk water retention through a more open and hydrated network structure.

**Figure 4:**
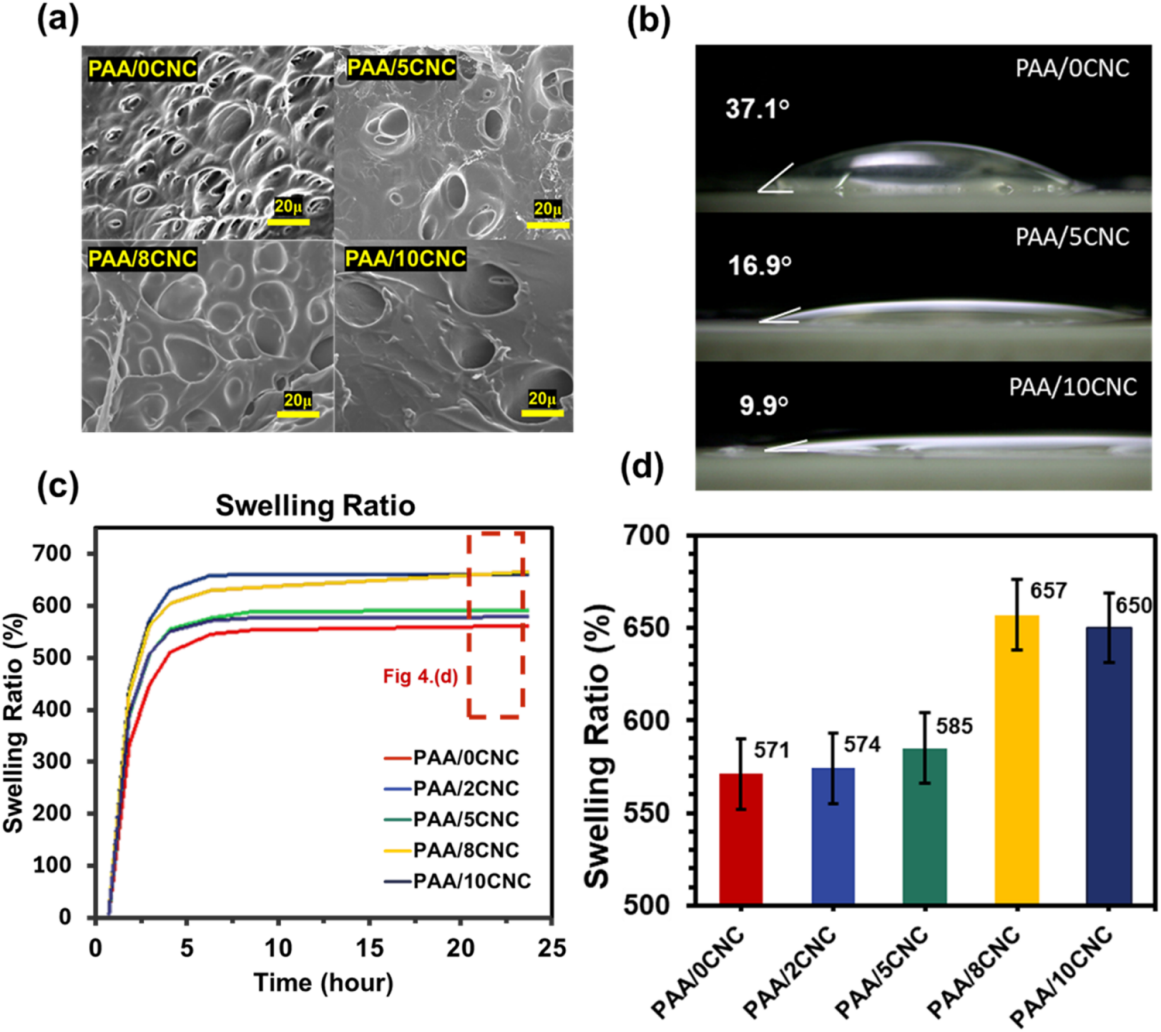
(a) SEM micrographs of PAA/x-CNC (b) Static contact-angle images for PAA/CNC hydrogels with increasing CNC loading. (c) Time-dependent swelling kinetics of representative PAA/CNC formulations. (d) Equilibrium swelling ratio (ESR) as a function of CNC content after 24 h.

The hydrophilic nature of PAA/CNC hydrogels is quantified by contact-angle and swelling measurements. CNC-free PAA films show a contact angle of 37.1°, whereas CNC incorporation lowers this value to 9.9° at 10 wt % CNC (Figure 4b), signifying near-complete wetting. This enhancement is attributed to hydroxyl-rich CNC surfaces that increase polarity and hydrogen-bonding interactions, consistent with intensified stretching of the O–H bond in the FTIR spectra (Figure 2).

Bulk water retention exhibits a parallel trend. The swelling ratio sharply increases with CNC loading, peaking at 657% at 8 wt % CNC after 24 h (Figure 4c,d). This improvement is attributed to the inherent hydrophilicity of CNCs and their function as nanoscale physical cross-linkers, which expands the free volume of the network. This expansion is reinforced by hydrogen bonding, thereby strengthening the polymer matrix. At a concentration of 10 wt % CNC, the formation of a denser, more tightly cross-linked network modestly restricts water ingress, leading to a slight reduction in swelling from the peak value. Although its swelling ratio is slightly lower than that of the 8 wt % sample, the difference lies within the acceptable standard deviation. As the CNC content increases, the pores become more visible and slightly larger, suggesting that CNC not only enhances hydrophilicity but also facilitates the formation of interconnected microvoids that favor water diffusion through the porous network. This is mainly because the abundant hydroxyl groups on CNC form strong hydrogen bonds with the polymer chains, locally disturbing the curing uniformity and inducing microphase separation, which in turn promotes the generation and expansion of pores within the matrix. This demonstrates a plateauing tendency above the 8% formulation, as the hydration capacity is maintained while simultaneously improving mechanical integrity. The synergistic effect of CNC loading and photocuring allows for fine-tuning both surface and bulk hydration, resulting in a hydrogel platform with a balance of water management and robust mechanical properties.

### 3.4 Sweat Monitoring with PAA/10CNC Hydrogel Coating

Based on the comparative data in Table 1, the PAA/10CNC hydrogel emerges as the most balanced and robust formulation for practical deployment. Among all compositions, it exhibits the lowest contact angle of 9.9°, which ensures excellent wettability and rapid liquid spreading across the surface. In terms of swelling behavior, the equilibrium swelling ratio of PAA/10CNC was measured to be approximately 650%, which exceeds those of the 0CNC, 2CNC, and 5CNC samples and is comparable to that of PAA/8CNC within acceptable standard deviation. From a mechanical perspective, the 10CNC sample achieves the highest maximum tensile strength of 180.7 kPa and the greatest Young’s modulus of 1.25 MPa, representing substantial improvements over the unfilled PAA matrix. These enhancements demonstrate that CNCs reinforce the hydrogel by both stiffening the network and increasing its load-bearing capacity. Although the gelation time increases progressively with CNC concentration and reaches 4 s at 10CNC, the curing process remains rapid enough for efficient UV processing and large-scale fabrication. Comprehensively, the combination of enhanced wettability, stable swelling behavior, and optimized mechanical properties clearly identifies PAA/10CNC as the most promising candidate for real-world sweat-sensing applications, as further supported by the electrochemical evaluations presented in Figures S5 and S6.

**Table 1:** Key Properties of the PAA/CNC Hydrogels

**formulation**	**contact angle** (°)	**swelling ratio** (%)	**max tensile strength** (kPa)	**Young’s modulus** (MPa)	**gelation time** (s)
PAA/0CNC	37.1	571	133	0.73	1.8
PAA/2CNC	27.1	574	144.8	0.84	2.5
PAA/5CNC	16.9	585	174.3	0.91	3
PAA/8CNC	11.0	657	178.8	1.03	3.7
PAA/10CNC	9.9	650	180.7	1.25	4

Electrochemical impedance spectroscopy (EIS)^24^ was employed to characterize the electrode–hydrogel interface using a Randles equivalent circuit, comprising solution resistance (*R*_s_), double-layer capacitance (*C*_dl_), charge-transfer resistance (*R*_ct_), and Warburg impedance (*Z*_w_). Through this model, EIS provided quantitative insight into the interfacial and transport phenomena governing sweat sensing. *R*_s_, which is inversely related to ionic conductivity (κ≈Λc), was shown to decrease systematically with increasing NaCl concentration (as shown in Figure 6c). The capacitive behavior of the electrode, reflected in *C*_dl_, was resolved from the semicircle curvature in Nyquist space, while *R*_ct_ was extracted from the semicircle diameter, indicating the kinetics of electron transfer across the interface (Figure S4). As shown in Figure 5, at low frequencies (e.g., 1 Hz), a distinct diffusion-related feature associated with *Z*_w_ was observed, reflecting the contribution of ion transport within the hydrogel matrix. Correspondingly, *R*_s_ was determined from the high-frequency intercept, *R*_ct_ from the semicircle diameter, and *C*_dl_ from the low-frequency response, as described by eq 2 (where ω = 2πf is the angular frequency):

2 $$Z\left(\omega \right)=R_{s}+\frac{1}{{j\omega C}_{\mathrm{dl}}+\frac{1}{R_{\mathrm{ct}}+Z_{\omega }}}$$

**Figure 5:**
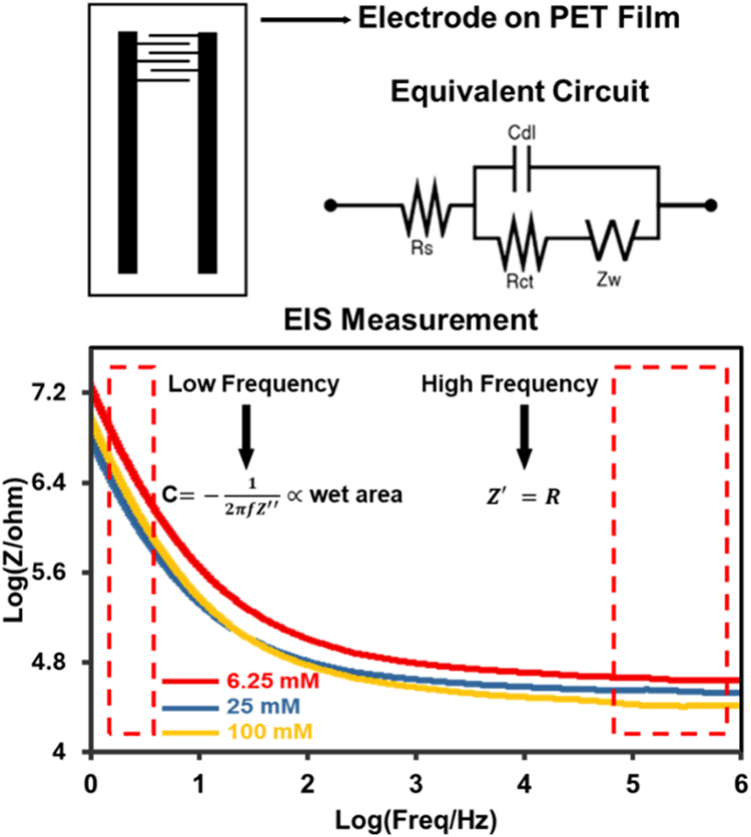
Equivalent circuit model of the electrode–hydrogel system used for EIS analysis.

Capacitive volume readout and resistive salinity detection are characterized on hydrogel-coated IDEs to assess signal quality. As shown in Figure 6a, uncoated IDEs yield only a transient capacitance spike that rapidly decays due to poor wettability and wicking into the textile, whereas PAA/10CNC-coated IDEs (optimized; Table 1) exhibit a robust, stepwise increase with successive 10 μL NaCl additions, accurately resolving incremental volume and demonstrating superior contact stability. Under a programmed flow rate profile (Figure 6b), capacitance follows the input in real time with <5% deviation and response times <30 s; the inset in Figure 6b shows a strong linear relationship between capacitance and flow rate. Having established reliable volume quantification, the salinity readout is then evaluated. The resistance decreases monotonically with increasing NaCl concentration from 6.25 to 100 mM (Figure 6c), consistent with enhanced ionic conductivity and faster charge transfer across the hydrated network. Stepwise additions (6.25 and then 25 mM) yield an intermediate equivalent (∼18 mM), capturing the expected dilution effect (Figure 6d). Concentration readout remains stable across varying flow rates (Figure 6c, inset), confirming independence from volumetric fluctuations. Finally, to verify suitability for prolonged use, long-term immersion tests show that hydrogel-coated electrodes retain >97% of initial capacitance after 7 days, with <3% signal fluctuation (Figure S9), underscoring robustness against evaporation and mechanical fatigue and addressing limitations of microfluidic or multilayer transport-based sweat sensors.

**Figure 6:**
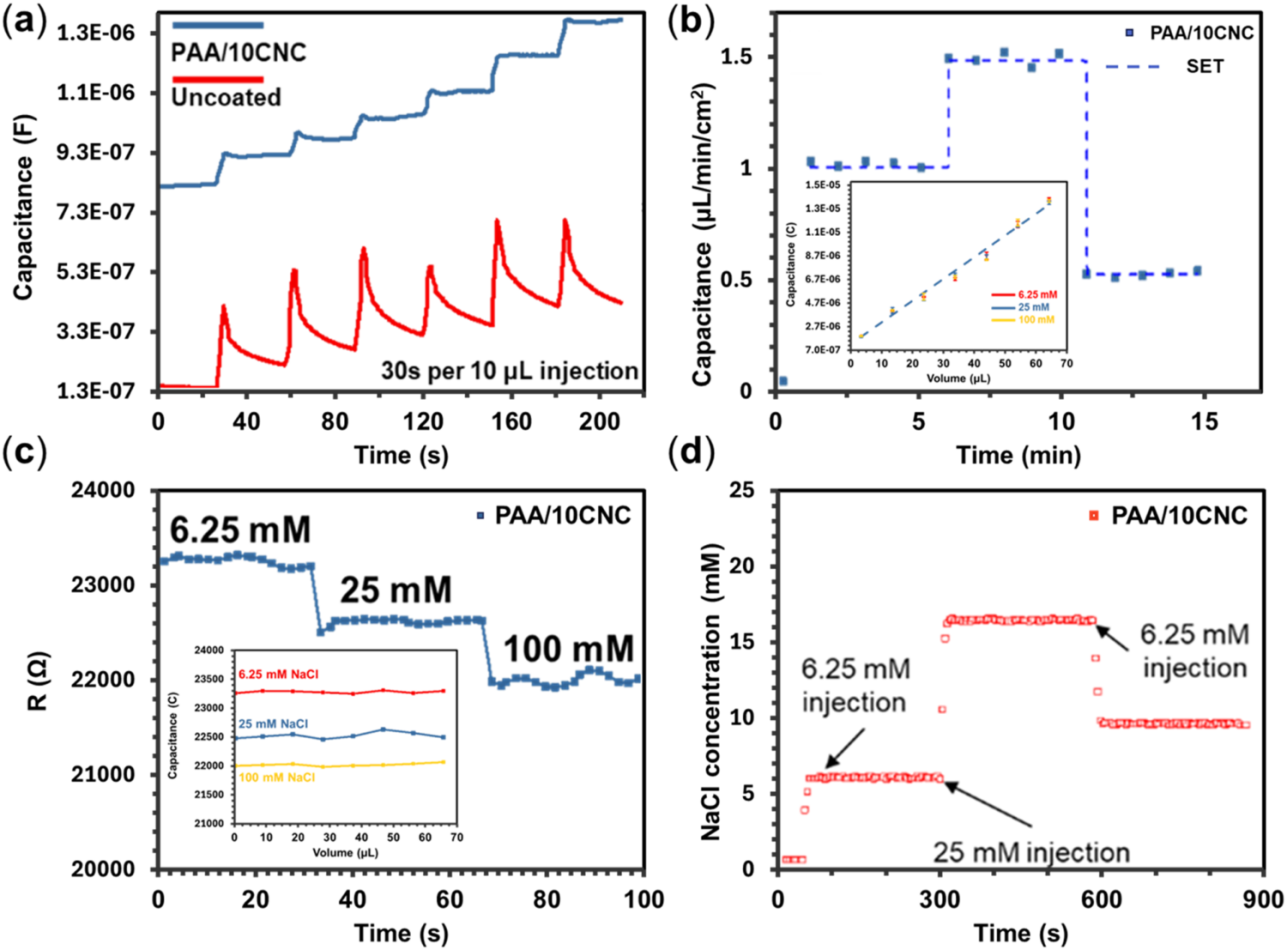
(a) Capacitance response to stepwise NaCl additions. (b) Capacitance tracking under a programmed flow rate profile. (c) Resistance as a function of the NaCl concentration. (d) Detection of sequential concentration steps with dilution effects.

### 3.5 Comparative Benchmark with Existing Sweat Sensing Systems

Performance of benchmark indicates that our photocurable PAA/CNC-based hydrogel sensor has representative state-of-the-art platforms, such as epidermal microfluidic patches, screen-printed textile sensors, and other hydrogel-based systems (Table 2).

**Table 2:** Comparative Benchmarking of Wearable Sweat-Sensing Platforms for Flow and Ion Monitoring

**platform**	**type of flow rate sensing**	**type of ion detection**	**response time**	**biocompatibility**	**durability**	**fabrication method**
this work (PAA/CNC)	capacitance	impedance	<30 s	excellent (PAA + 1173)	>1 week soaking	UV curing
Gao et al.^5^ (Nature, 2016)	microfluidics	colorimetric and EC	∼1 min	high (PDMS/PE)	≥2 h, single-use	soft lithography
Bandodkar et al.^15^ (Sci. Adv., 2019)	electrochemical textile	electrochemical	<60 s	high (screen-printed)	20 washing cycles	screen-printing
Yin et al.^7^ (Nat. Electron., 2022)	standalone sensing and display	EC sensors: glucose, lactate, Na^+^, pH	<1 s	excellent (stretchable SEBS-based)	1500 cycles at 20% strain	PEDOT: PSS ECDs
Binabaji et al.^25^ (Anal. Chem., 2024)	VOC-focused	fluorometric detection of acetone, ammonia	∼2–5 min	excellent (gelatin/PVA hydrogel)	high	paper-based microfluidics + smartphone
gatorade patch (commercial)	colorimetric	no	∼5 min	moderate	disposable	proprietary

## 4 Device Integration and Real-Time Sweat Monitoring

To ensure wearable compatibility and practical deployment, the PAA/CNC-coated interdigitated electrode (IDE) is laminated onto a fabric substrate (Figure 7). The IDE is first coated with the hydrogel layer and then bonded to a heat-fusible, double-sided adhesive film that is precision-cut to the electrode outline for accurate alignment. The stack is subsequently heat-pressed onto the fabric, yielding a compliant, stretchable, and skin-conformal interface that maintains contact during motion. To bolster mechanical stability, a 1 × 1 cm reinforcement film is added as a local anchor, distributing strain and reducing the risk of motion-induced delamination. Electrical interconnection is provided by a flexible ribbon cable, which offers inherent strain relief and routes signals to data-processing chips embedded in a wearable headband. This integration strategy stabilizes the sensor–fabric coupling and secures the hydrogel interface under dynamic conditions, enabling reliable real-time sweat collection and electrochemical analysis.

**Figure 7:**
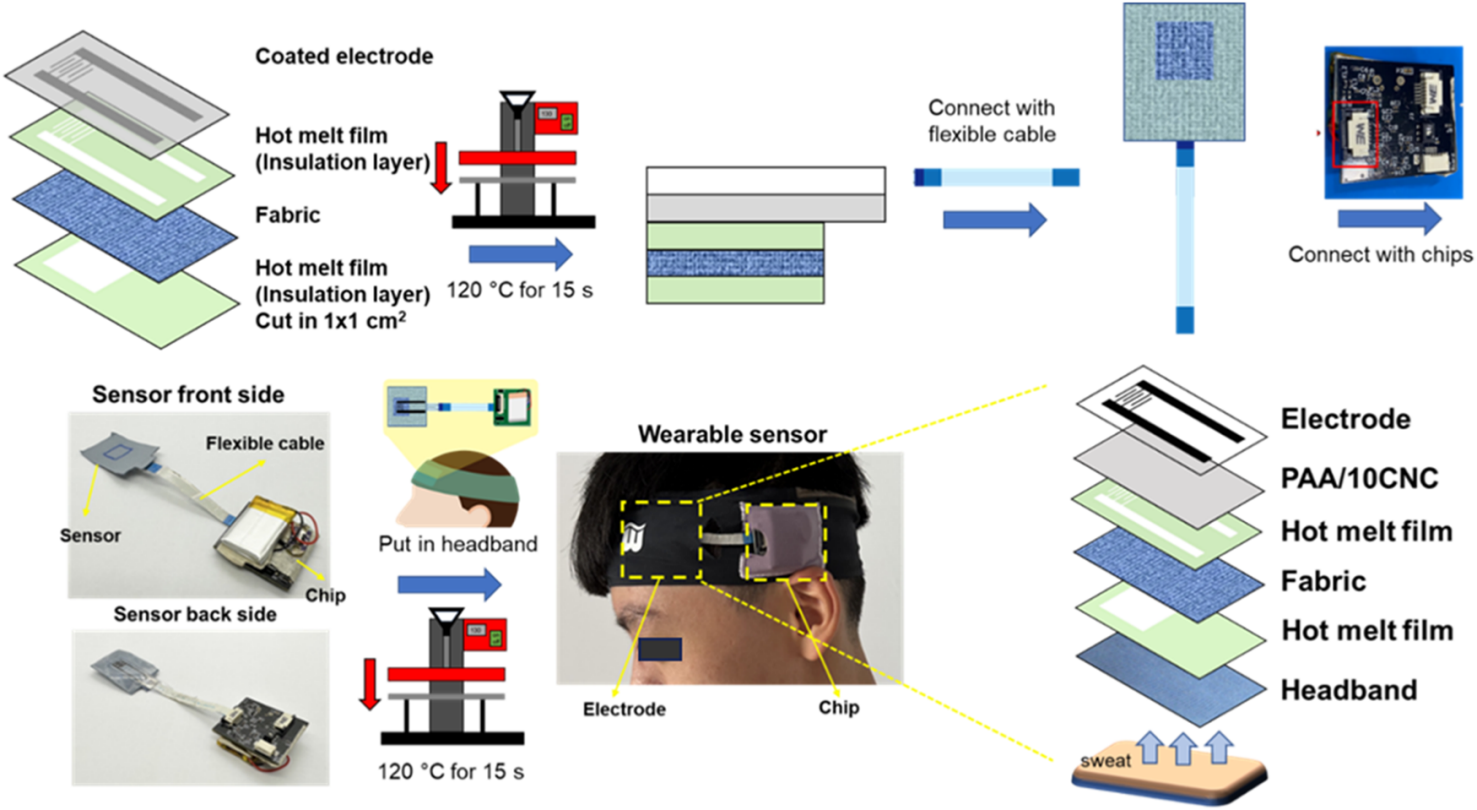
Device integration and electrode design of smart sweat-sensing headband.

Real-time monitoring is evaluated in a calibrated dynamic test (precalibration shown in Figures S5 and S6). As illustrated in Figure 8a,b, the protocol comprises three phases designed to perturb both sweat rate and composition: running at 8 km h^–1^ for 10 min, increasing to 10 km h^–1^ for 10 min, and then walking after ingesting 300 mL of water. During the workload escalation, capacitance (a proxy for wetted area/film thickness and thus sweat rate) increases while resistance shifts consistently with rising ionic strength, reflecting the thermoregulatory drive to dissipate heat via eccrine secretion. Following hydration and reduced exertion, capacitance falls, and the resistance response reverses toward higher values, indicating lower sweat output and dilution of the ionic content. This rate–composition coupling aligns with known physiology: higher glandular flux reduces ductal reabsorption and elevates sweat ionic concentration, whereas oral hydration lowers osmotic drive and promotes more dilute sweat. Together, these trends demonstrate continuous, stimulus-responsive tracking of both the sweat rate and ionic composition in real time.

**Figure 8:**
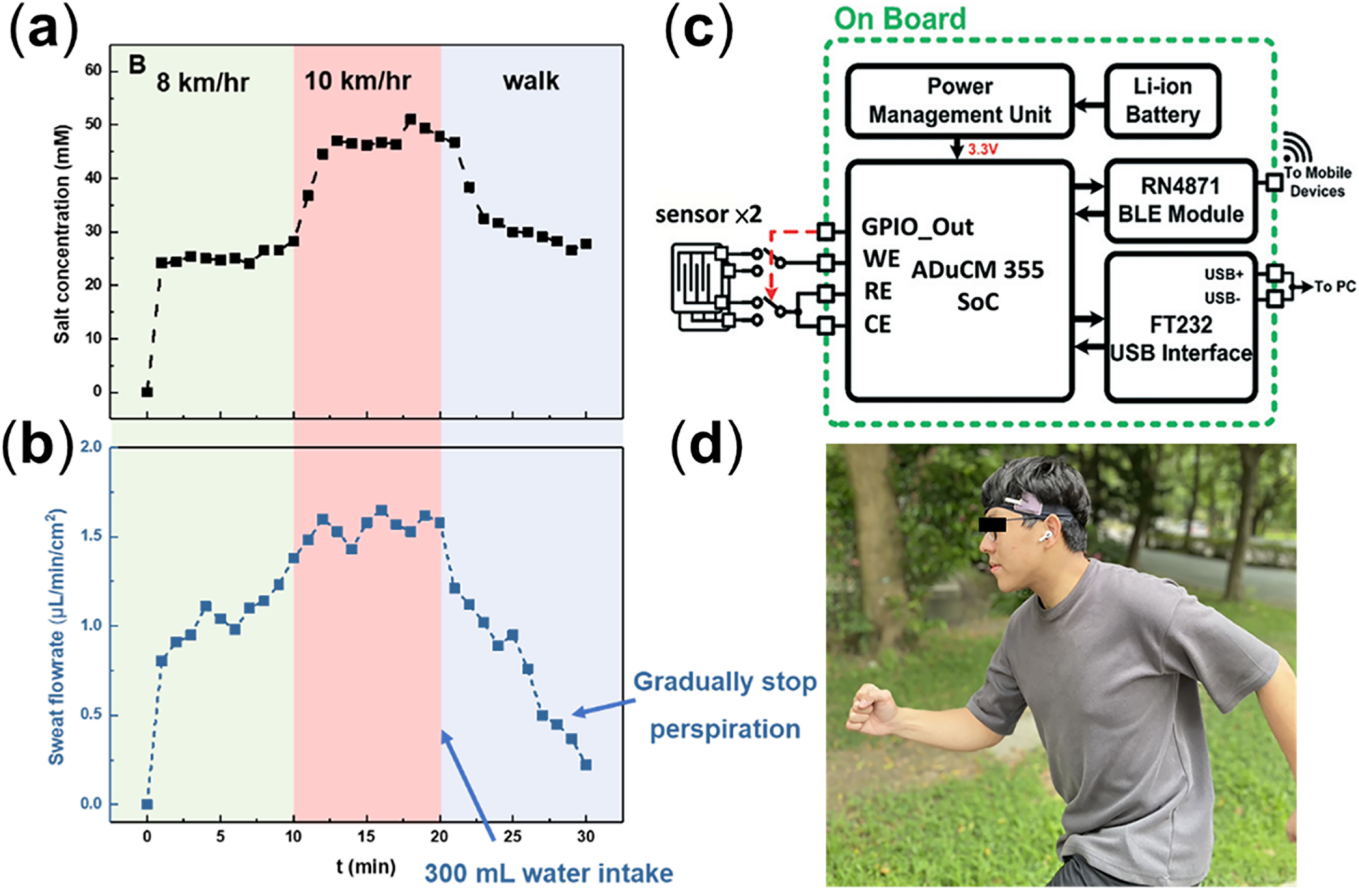
Real-time sweat monitoring with the smart hydrogel sensor system. (a) Resistance (ion concentration) and (b) capacitance (sweat rate) during dynamic activity. (c) Schematic of the wireless sensing device architecture. (d) On-body demonstration of practical sweat monitoring.

The sensing modality is implemented via electrochemical impedance spectroscopy (EIS) on the ADuCM355 system-on-chip (Figure 8c). Sinusoidal excitation applied to the hydrogel-based interface yields magnitude/phase responses that are converted to impedance and mapped to an equivalent circuit, enabling separation of solution resistance (ion concentration) and interfacial/capacitive terms (sweat film dynamics). For data transfer, an FT232 USB interface provides a wired readout, while an RN4871 Bluetooth Low Energy module supports wireless streaming, enabling continuous, untethered monitoring (Figure 8d). Taken together, the closed-loop calibration, physiologically consistent signal trajectories, and robust telemetry indicate that the smart hydrogel sensor is well suited for on-body, real-time sweat analytics in wearable health applications.

## 5 Conclusion

In this study, a UV-curable PAA/CNC hydrogel is developed for wearable sweat sensors, offering fast curing, high swelling, good strength, and durable wet stability. Incorporation of CNC into PAA polymers improves structural integrity and hydrophilicity and mitigates degradation-related issues commonly observed in conventional PAA hydrogels. Rheological analysis confirms consistent cross-linking across CNC loadings, while FTIR and SEM verify esterification and the formation of a porous network. 10 wt % CNC formulation specifically maximizes stiffness and strength while maintaining good surface wettability and swelling ratio, delivering a balanced combination of hydrophilicity and structural stability. For practical integration, the optimized hydrogel is applied to a textile-based interdigitated electrode and interfaced with an embedded ADuCM355 system-on-chip featuring Bluetooth and USB modules for real-time acquisition and wireless transmission. Electrochemical tests demonstrate dual functionality—sweat flow rate via capacitance and ion concentration via resistance—with rapid response (<30 s) and minimal drift (<5% error). Long-term immersion further highlights wetting stability, with consistent performance maintained for over 7 days. Compared with systems that rely on multilayer encapsulation, rigid electronics, or unstable sensing films, this monolithic, flexible design offers a more practical, skin-conformable solution. A headband-integrated prototype successfully monitors real-time sweat dynamics during physical activity, confirming the platform’s potential for continuous physiological tracking. Overall, the approach provides a scalable, low-cost, and biocompatible route to IoT-enabled wearable health monitoring, with applications in personalized diagnostics, athletic performance optimization, and responsive smart textiles.

## Supplementary Material



## Notes

Glass transition temperature analysis of PAA/CNC hydrogel films; electrochemical impedance analysis describing solution resistance, double-layer capacitance, charge-transfer resistance, and Warburg impedance; demonstration of capacitance variation with wetted area under low-frequency conditions; demonstration of resistance dominance in the high-frequency regime; calibration of capacitance response with incremental NaCl additions; calibration of resistance response to stepwise NaCl concentration changes; and long-term wetting stability test of PAA/10 wt % CNC hydrophilic films compared with pure CNC films (PDF)
