# Supplementary material for: Photocurable Nanocellulose-Based Hydrogel for Real-Time Electrochemical Sweat Monitoring in Smart Textiles
Source: Langmuir. 2025 Dec 15;41(51):34474–83. doi: 10.1021/acs.langmuir.5c04977 (PMC12756917; doi:10.1021/acs.langmuir.5c04977)
Supplement: Supplementary file 1 [file la5c04977_si_001.pdf]

# Supporting Information

# Photocurable Nanocellulose-Based Hydrogel for Real-Time Electrochemical Sweat Monitoring in Smart Textiles

*Chuang, Kai-Wen†, Li, Po-Kuan†, Huang, Yun-An† and Liao, Ying-Chih*†*

†Department of Chemical Engineering, National Taiwan University, Taipei, Taiwan 10617

**Corresponding author: Liao, Ying-Chih*

*E-mail:* liaoy@ntu.edu.tw

**Figure S1.** Thermal analysis of PAA/CNC hydrophilic film...........................................3

**Figure S2.** Rheological and Mechanical Characterization of PAA/CNC Hydrogels (a) Storage (G′) and loss modulus (G″) evolution of PAA/CNC hydrogels under UV exposure ..........................................................................................................................4

**Figure S3.** Tensile stress–strain profiles of PAA/CNC hydrogels with varying CNC formulations. ........................................................................................................5

**Figure S4.** Nyquist plots of PAA/0CNC and PAA/10CNC hydrophilic film. ..................7

**Figure S5.** Capacitance responses of PAA/xCNC hydrogel films under successive additions of NaCl solutions with different flowrate. ........................................................8

**Figure S6.** Resistance responses of PAA/xCNC hydrogel films under successive additions

of NaCl solutions with different concentrations. ................................................................9
**Figure S7.** Calibration curve of capacitance vs. flow rate in varying flowrate. ...............10
**Figure S8.** Calibration curve of Resistance vs. Time in varying concentration. .............10
**Figure S9.** Wetting stability test showing capacitance retention over time and under soaking conditions. ..........................................................................................................11

Thermal analysis was performed to evaluate the glass transition temperature (Tg) of the photocurable PAA/CNC hydrogel films, as shown in **Figure S1**. The Tg represents a critical parameter in determining the polymer's operational stability, as it defines the boundary between glassy and rubbery states of the material. In this study, the measured Tg of the PAA/CNC films were found to be significantly higher than the typical physiological and environmental operating conditions of wearable devices. This elevated Tg indicates that the hydrogel matrix remains in a viscoelastic yet structurally stable regime during use, thereby preventing premature softening or mechanical failure under body temperature fluctuations.

Furthermore, the high Tg supports the durability of the hydrogel during cyclic loading and deformation, complementing the stress relaxation measurements that revealed extended relaxation times in CNC-reinforced samples. The combination of a stable glass transition far above room and body temperatures, together with the viscoelastic adaptability introduced by CNC incorporation, ensures that the PAA/CNC hydrogel maintains both its structural integrity and functional responsiveness during long-term operation. These results confirm that the thermal robustness of the hydrogel network is a key contributor to its

reliability as a skin-conformal interface for wearable sweat sensing applications.

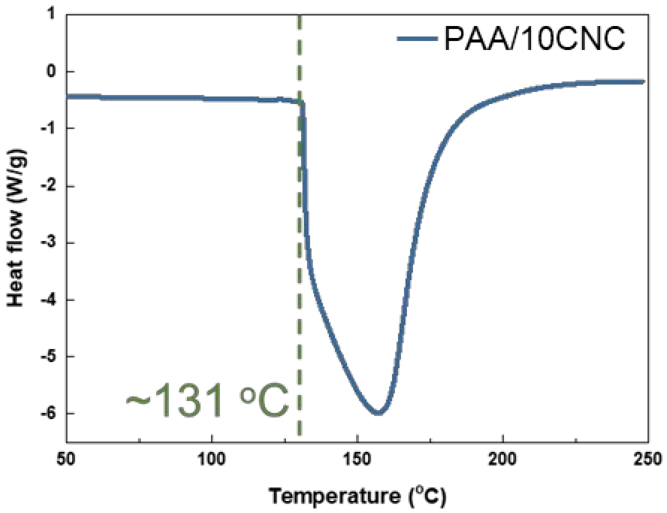


**Figure S1.** Thermal analysis of PAA/CNC hydrophilic film

**Figure S2.** shows the rheological and mechanical behavior of PAA/CNC hydrogels at different CNC concentrations. Upon UV irradiation at the 20th second, both G′ and G″ increase sharply within a few seconds, confirming ultrafast photopolymerization and network formation. Although higher CNC content accelerates gelation, the storage modulus slightly decreases after curing due to enhanced viscoelastic relaxation and shear-thinning behavior, as shown in Figure 3. The Young's modulus, however, increases with CNC loading since the rigid CNC domains reinforce the cured matrix under uniaxial compression.

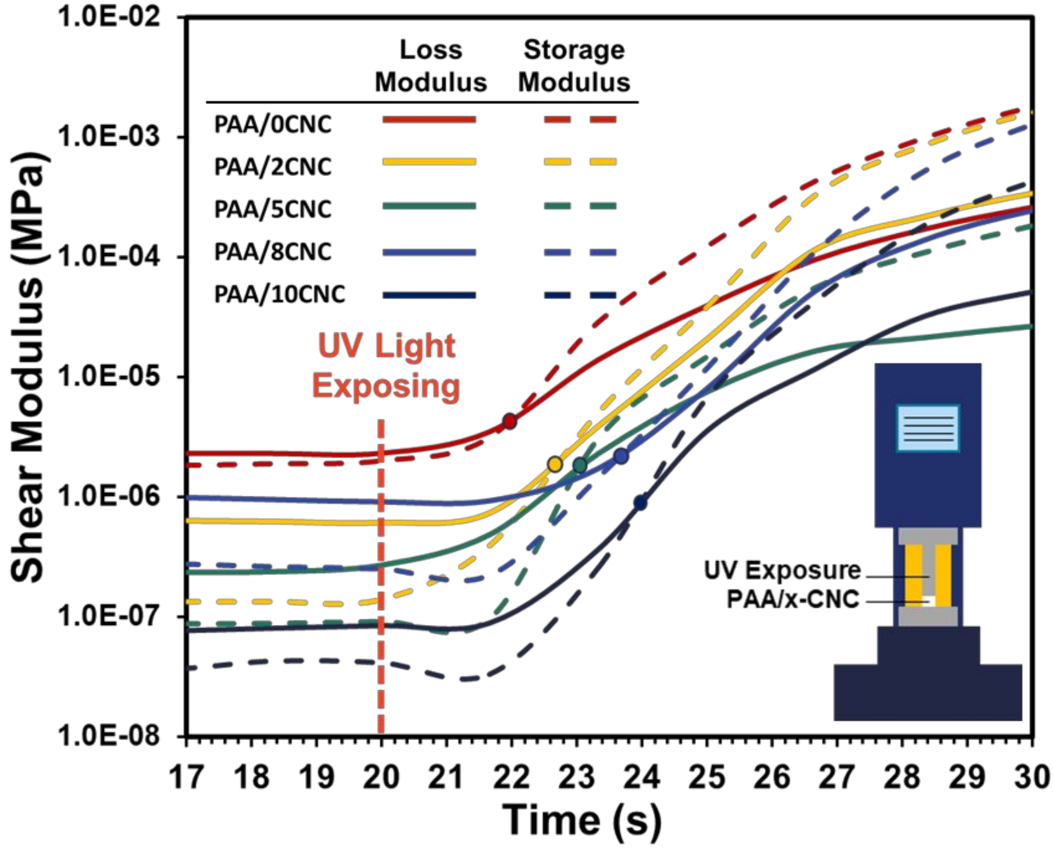


**Figure S2.** Rheological and Mechanical Characterization of PAA/CNC Hydrogels (a) Storage (G′) and loss modulus (G″) evolution of PAA/CNC hydrogels under UV exposure

**Figure S3** further corroborates this trend through tensile testing, where hydrogels with elevated CNC levels exhibit higher tensile strength and improved mechanical resilience. Together, these results confirm that CNC incorporation not only strengthens the hydrogel matrix but also stabilizes its structure during mechanical deformation.

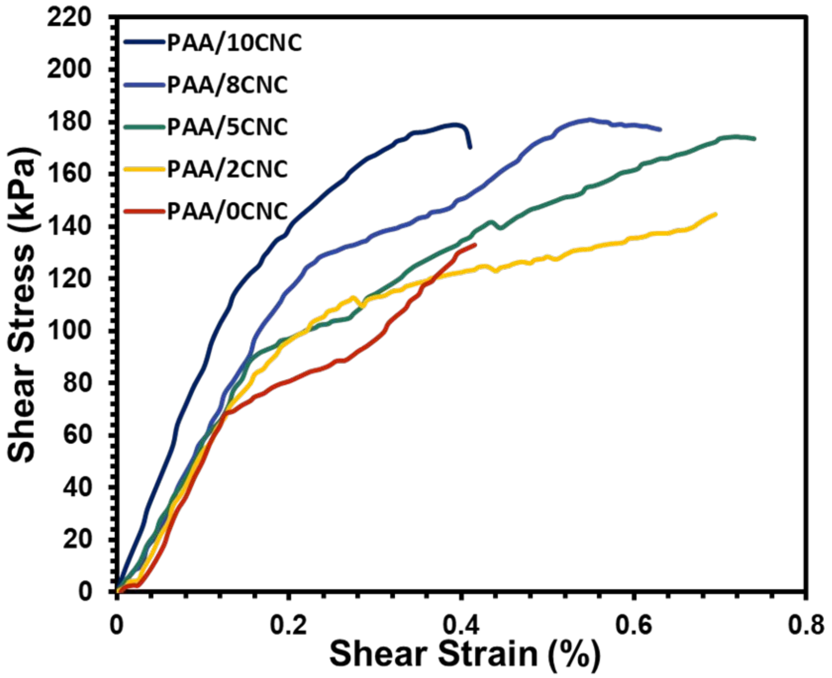


**Figure S3.** Tensile stress–strain profiles of PAA/CNC hydrogels with varying CNC formulations.

In **Figure S5**, Nyquist plots illustrating the electrochemical behavior of PAA hydrogel interfaces with and without CNC incorporation, highlighting key impedance features for subsequent monitoring analyses. The electrode-hydrogel interface, modeled by the Randles equivalent circuit[1], intricately captures the electrochemical dynamics at play in sweat-sensing applications through its components: solution resistance (Rs), double-layer capacitance (Cdl), charge-transfer resistance (Rct), and Warburg impedance (Zw). Rs reflects ohmic losses in the bulk electrolyte, governed by equation S1:

$$\mathrm{Rs} = \frac{\mathrm{L}}{\kappa \mathrm{A}} \qquad (\mathrm{S1})$$

, where $\kappa \approx \Lambda c$ (with $\Lambda$ as the molar conductivity, approximately 0.0126 $S \cdot m^2/mol$ for dilute NaCl, and c as concentration in $mol/m^3$), decreasing due to enhanced ionic conductivity

with higher NaCl levels, as evidenced by the descending impedance plateaus in Bode magnitude plots at high frequencies.

Cdl, modeled as equation S2:

$$C_{dl} \approx \frac{\epsilon A_w}{d} \quad (S2)$$

(where $\epsilon$ is the permittivity, Aw the wetted electrode area, and d the double-layer thickness, shrinking with increasing ionic strength as $d \propto \frac{1}{\sqrt{c}}$), contributes to the capacitive semicircle in Nyquist plots, its magnitude increasing with wetting Area.

Rct, derived from the Butler-Volmer equation (equation S3) as:

$$R_{ct} = \frac{RT}{nFi_0A} \quad (S3)$$

(where $i_0$ is the exchange current density), represents kinetic barriers to electron transfer and forms the semicircle diameter, typically around 1 kΩ in inert systems.

Zw, expressed as equation S4:

$$Zw = \frac{\sigma}{\sqrt{\omega}}(1 - j) \quad (S4)$$

with $\sigma = \frac{RT}{\sqrt{2}n^2F^2Ac\sqrt{D}}$. Warburg element produces equal-magnitude real and negative imaginary impedance components that both grow as frequency decreases, a signature of diffusion-limited $Na^+/Cl^-$ transport with an inverse square-root trend.

As shown in the Nyquist plots, PAA/10CNC exhibits a significantly smaller semicircle and shorter diffusion tail compared to the CNC-free PAA sample. This indicates a much lower interfacial resistance and faster ion transport across the hydrogel–electrode interface. The incorporation of CNC enhances ionic mobility and charge transfer efficiency, confirming

that the 10CNC formulation possesses the most favorable electrochemical characteristics among all samples.

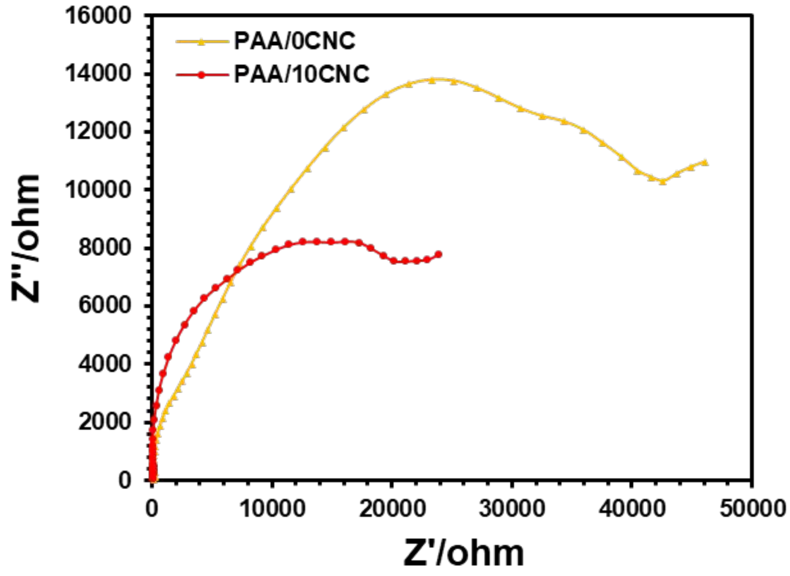


**Figure S4.** Nyquist plots of PAA/0CNC and PAA/10CNC hydrophilic film.

In **Figure S6**, the PAA/10CNC film exhibits the highest double-layer capacitance (Cdl) among all formulations measured under a 6.25 mM NaCl electrolyte, reflecting its enhanced ionic conductivity and interfacial charge storage capability. This improvement is attributed to the increased hydrophilicity and fluid-retention capacity provided by higher CNC content, which facilitates faster ion migration within the hydrogel matrix. The clear rise in Cdl for PAA/10CNC confirms its efficient electrolyte accessibility and superior electrochemical responsiveness compared to other formulations.

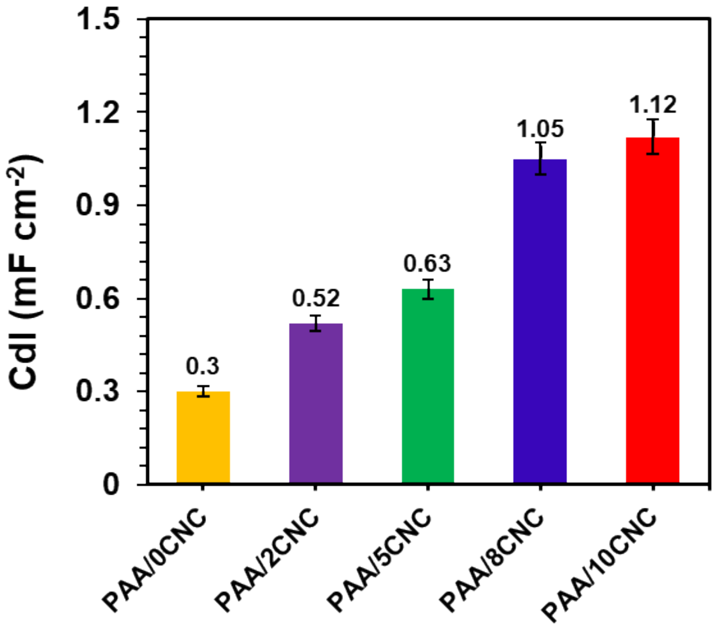


**Figure S5.** Capacitance responses of PAA/xCNC hydrogel films under successive additions of NaCl solutions with different flowrate.

**Figure S7** presents the resistance values (Rs) of PAA/xCNC hydrophilic films measured under a 6.25 mM NaCl electrolyte. The overall resistance decreases systematically with increasing CNC content, reflecting the enhanced ionic conductivity and continuous charge transport pathways provided by CNC incorporation. Among all samples, PAA/10CNC exhibits the lowest resistance (~22500 Ω), confirming its superior ionic conduction and rapid interfacial response. This improvement arises from the combined effects of higher hydrophilicity, greater electrolyte affinity, and efficient ion migration within the CNC-rich network.

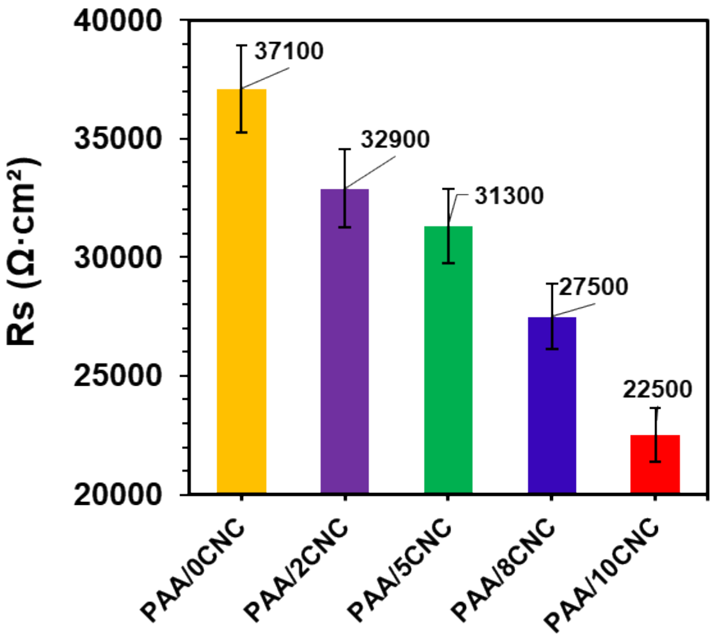


**Figure S6.** Resistance responses of PAA/xCNC hydrogel films under successive additions of NaCl solutions with different concentrations.

To ensure the viability of capacitance- and resistance-based sensing prior to the deployment of the PAA/CNC hydrogel films, preliminary calibration tests were conducted. **Figure S8** presents the capacitance response under incremental NaCl additions, showing a linear increase in capacitance with fluid volume ($R^2 > 0.98$), which verifies the reliability of capacitance as a proxy for liquid volume detection. **Figure S9** displays the resistance response to stepwise changes in NaCl concentration, where distinct decreases in resistance were observed with each increase in electrolyte concentration. This stepwise trend confirms that resistance measurements can effectively resolve ionic strength variations in aqueous environments. Together, these calibration studies validate that both capacitance and resistance signals are stable, reliable, and directly correlated with liquid volume and salinity, providing a solid foundation for their subsequent application in the PAA/CNC hydrogel–based real-time sweat monitoring system.

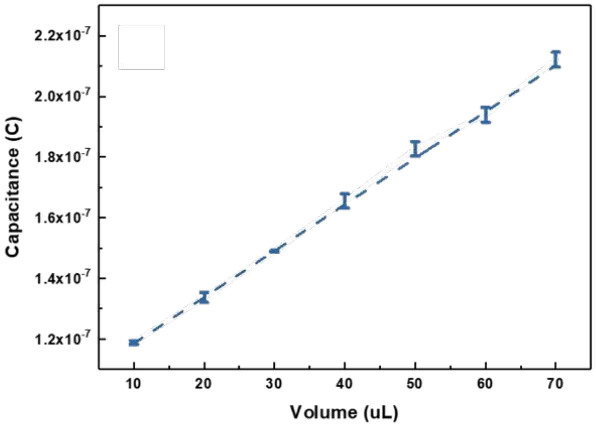


**Figure S7.** Calibration curve of capacitance vs. flow rate in varying flowrate.

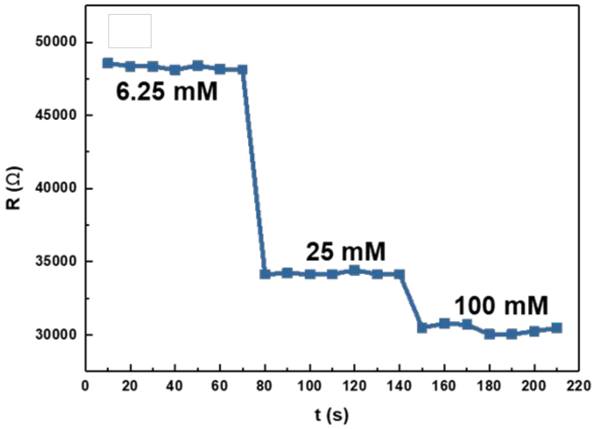


**Figure S8.** Calibration curve of Resistance vs. Time in varying concentration.

**Figure S9** illustrates the long-term wetting stability of the PAA/10 wt% CNC hydrophilic film by monitoring its capacitance after one week of storage. In sharp contrast to the pure CNC film, which underwent significant degradation due to the absence of PAA cross-linking, the PAA/10 wt% CNC composite film exhibited a remarkably stable capacitance signal. This minimal fluctuation, with a variation of less than 3%, demonstrates the robust long-term stability and enhanced resistance to degradation afforded by the PAA matrix.

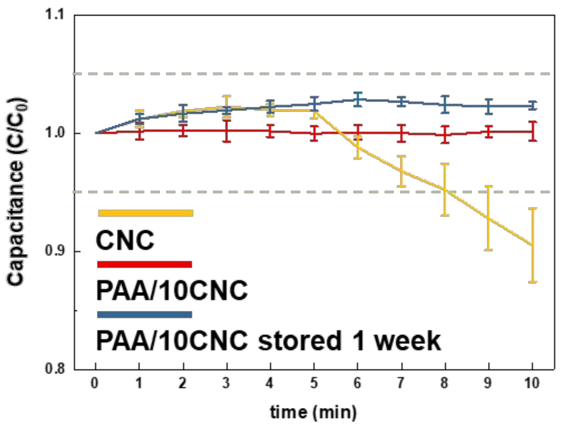


**Figure S9.** Wetting stability test showing capacitance retention over time and under soaking conditions.

## Reference

(1) Vyas, R. N.; Li, K.; Wang, B. Modifying randles circuit for analysis of polyoxometalate layer-by-layer films. *The Journal of Physical Chemistry B* **2010**, *114* (48), 15818-15824.
